# Impact of TiO_2_ Nanostructures on Dye-Sensitized Solar Cells Performance

**DOI:** 10.3390/ma14071633

**Published:** 2021-03-26

**Authors:** Paweł Gnida, Paweł Jarka, Pavel Chulkin, Aleksandra Drygała, Marcin Libera, Tomasz Tański, Ewa Schab-Balcerzak

**Affiliations:** 1 Centre of Polymer and Carbon Materials, Polish Academy of Sciences, 34 M. Curie-Skłodowska Str., 41-819 Zabrze, Poland; pgnida@cmpw-pan.edu.pl; 2 Institute of Engineering Materials and Biomaterials, Silesian University of Technology, 18a Konarskiego Str., 44-100 Gliwice, Poland; pawel.jarka@polsl.pl (P.J.); aleksandra.drygala@polsl.pl (A.D.); 3 Faculty of Chemistry, Silesian University of Technology, 9 Strzody Str., 44-100 Gliwice, Poland; pavel.chulkin@polsl.pl; 4 Institute of Chemistry, University of Silesia, 9 Szkolna Str., 40-007 Katowice, Poland; marcin.libera@us.edu.pl

**Keywords:** DSSC, TiO_2_ nanostructures, N719, electrochemical impedance spectroscopy

## Abstract

The effect of TiO_2_ nanostructures such as nanoparticles, nanowires, nanotubes on photoanode properties, and dye-sensitized solar cells photovoltaic parameters were studied. The series of dye-sensitized solar cells based on two dyes, that is, commercially N719 and synthesized 3,7′-bis(2-cyano-1-acrylic acid)-10-ethyl-phenothiazine were tested. Additionally, the devices containing a mixture of this sensitizer and chenodeoxycholic acid as co-adsorbent were fabricated. The amount of adsorbed dye molecules to TiO_2_ was evaluated. The prepared photoanodes with different TiO_2_ nanostructures were investigated using UV-Vis spectroscopy, optical, atomic force, and scanning electron microscopes. Photovoltaic response of constructed devices was examined based on current-voltage characteristics and electrochemical impedance spectroscopy measurements. It was found that the highest UV-Vis absorption exhibited the photoanode with nanotubes addition. This indicates the highest number of sensitizer molecules anchored to the titanium dioxide photoanode, which was subsequently confirmed by dye-loading tests. The highest power conversion efficiency was (6.97%) for solar cell containing nanotubes and a mixture of the dyes with a co-adsorbent.

## 1. Introduction

Since the introduction by O’Regan and Grätzel in 1991, dye-sensitized solar cells (DSSCs) have been intensively investigated as promising candidates for next-generation solar cells because of their high photovoltaic performance, low production cost, and low environmental impact [1,2,3,4]. DSSCs are produced in fast, simple, and economical processes and their power conversion efficiency (PCE) reaches 14% [5,6,7,8]. Furthermore, DSSCs’ power output is relatively stable in various lighting conditions, including light emmiting diode (LED) and fluorescent light (as opposed to silicon solar cells) [9]. However, the PCE (depending on charge collection efficiency, light scattering ability, and charge recombination rate) and time of effective operation of DSSCs are still lower than those of silicon cells, limiting their widespread use. [10,11]. These parameters are closely related to the DSSCs photoanode properties [12]. Thus, the design and production of photoanodes with increased dye loading, light scattering, and charge transportability with a simultaneously reduced recombination reaction is one of the basic tasks required to achieve high PCE. The photoanodes are fabricated mostly from metal oxides such as TiO_2_, ZnO, Nb_2_O_5_, GaAs, CdS, SnO_2_ [6,7,8,9,13,14,15] in the form of mesoporous structures. Due to the large surface area, chemical long-term stability, little toxicity, large catalyst band edges, and low recombination rate, TiO_2_ is the most popular semiconductor material for DSSC photoanodes [10]. Nevertheless, mesoporous structures may contain large surface sites, which work like recombination points at the interface of the conductive layer and electrolyte, thereby lowering PCE [16]. However, in addition to nanostructures containing metal atoms, there are reports in the literature of the use of nanostructures such as carbon nanotubes, whose use has a direct impact on the photovoltaic performance of the solar cells being prepared. The presence of carbon nanotubes in sufficient quantity causes a significant increase in the charge accumulation on the photoanode [17].

One of the ways to increase the efficiency of newly developed DSSC is based on increasing the interaction between light transmission through an electrode with the dye molecules. This method is based on the use of materials with an increased light scattering in the photoanode structure, which increases the travel time of the light beam through the photoanode and improves the interaction of incident photons with dye particles [18]. One of the methods of obtaining materials with described properties is the use of one-dimensional materials such as nanorods [18], nanowires [19,20], nanotubes [21,22], nanofibers [23,24], and nanospindles [25,26]. However, these scattering layer materials have insufficient light adsorption due to their relatively small specific surfaces. The solution to this problem is therefore the production and development of multifunctional materials combining the desired properties of light diffusion with high dye adsorption such as hybrid layers that connect one- (1D), two- (2D), and three-dimensional (3D) nanostructures [27,28]. Combining such types of nanostructured phases have been intensively studied to increase surface area for dye adsorption (widening the optical length of the incident light) as well as enhance the diffusion of the electrolyte through the electrode pores and create direct pathways for fast collection of photogenerated electrons [29,30,31]. In addition to modifying the mesoporous layer itself, a blocking layer is added between the substrate, which is fluorine doped tin oxide (FTO), and the oxide layer. The blocking layer is usually made of a metal oxide such as ZnO. Its main function is to improve the contact between the FTO substrate and the mesoporous layer and to block unwanted charge flow. [32] 

It should be noted that the use of this type of hybrid material affects the trapping and de-trapping phenomena occurring in interfacial states and grain boundaries of photoanode NPs material, as well as reduces the occurrence of disordered aggregation and asymmetric networks at the grain boundaries.

Thus, the photoanodes combining hybrid nanostructures of 1D and 3D nanostructures have attracted attention due to the streamlined electron transport, efficiency of the charge collection, and relatively low recombination rate [33,34,35]. However, the main problem in DSSCs based on nanowire/nanorod-based is the inadequate surface area for dye loading and hence insufficient light scattering ability [36,37]. The problem could be solved by the addition of the TiO_2_ electrospun nanofibers/nanoparticles combination, which improves charge transport as well as light scattering with no loss in dye loading. This DSSC structure demonstrates higher efficiency than those made of pure TiO_2_ nanoparticles (NPs) [38]. Nowadays, numerous works of DSSC based on TiO_2_ 1D/3D hybrid nanostructures consider the connection of the dye loading with surface roughness and control it by changing the particle size and length of 1D nanostructures. Liu et al. in their work de-voted to translucent TiO_2_ nanowires (NWs)/ nanotubes (NTs) prepared by a two-step anodization method [39] demonstrate that TiO_2_ nanotube arrays could be successfully used in DSSCs. Hegazy et al. [40,41] comprehensively studied room temperature synthesis of TiO_2_ nanoparticles for DSSCs with liquid electrolyte. Zhao et al. describes TiO_2_ nanorod-flowers (NRFs) photoanode layers composed of 1D TiO_2_ nanorods array (NRA) and 3D TiO_2_ nanoflowers (NFs) of the efficiencies in the range 5.54–8.41%, [42]. The 8.38% of efficiency was obtained in the DSSC hybrid structure, including 3D anatase TiO_2_ architecture consisting of vertically aligned 1D hierarchical TiO_2_ nanotubes with ultra-dense branches and zero-dimensional (0D) hollow TiO_2_ microspheres with a rough surface [43]. The DSSC based on TiO_2_ nanofibers allowed to obtain an efficiency of 8.15% [24]. The application of TiO_2_ NPs in hierarchical spherical architecture increased the efficiency up to 9.35% [44]. The efficiency of 10.3% was obtained by Wang et al. for DSSC based on TiO_2_ photoanode with nanorod arrays etched in a secondary hydrothermal process with the use of hydrochloric acid [45].

Improving the dye adsorption through photoanodes architecture development is closely related to the development of new dyes. The development of new photoanodes with increased dye absorption and charge transfer, while at the same time extending the absorption of incident photons and injection of electrons into the semiconductor conduction band, is the way to obtain the highest efficiency of DSSC systems [45]. Currently, from the point of view of used dyes, the DSSCs can be categorized as based on metal-containing sensitizer (usually commercial, such as N3, N719, N749 based on Ru or YD2 with Zn [46,47,48]) and based on newly developed metal-free dye [49,50,51,52,53,54,55].

It should be noted that the manufacturing of metal-containing dyes is expensive and time-consuming and requires a complicated purification process [56,57,58,59]. Intensive work is underway on an alternative for metal-dyes, organic sensitizers. The use of the metal-free dyes reduces the cost of DSSCs production in comparison to metal-based dyes DSSCs with slightly lower efficiency [60,61,62]. The important parameters in the development of this kind of dye are: high solubility in solvents, high molar coefficient, wide range of light absorption, high resistance against photodegradation and photocorrosion, electrochemical and thermal stability, and frontier levels of molecular orbital energy aligned [63,64,65,66]. Research has confirmed that using two or more sensitizers in a co-sensitization system is a highly effective solution for light-harvesting efficiency in DSSCs [51,60]. Moreover, co-sensitization with organic dyes allows to reduce the amount of metal-containing dyes N719 dye applied and may reduce the cost of device preparation [57,65]. It should be also stressed that co-sensitization may improve the photovoltaic performance of solar cells [60].

Considering the above, the authors focused on innovative rapid and economical construction of photoanode with high-dimensional and structural repeatability, enabling accurate comparative studies between cells with different TiO_2_ nanocomponents in the future. The aim of such a solution was the manufacturing and study of DSSCs employing photoanode in the form of a hybrid structure combining nanoparticles with nanowires and nanotubes to improve the efficiency of the charge collection. In addition, the article presented the application of the fabricated photoelectrodes in new DSSC construction with synthesized 3,7′-bis(2-cyano-1-acrylic acid)-10-ethyl-phenothiazine dye, and with a commercial reference dye. It discusses utilizing the combination of an innovative photoanode construction with the simultaneous use of a synthesized dye, which is the way to develop modern DSSC constructions.

## 2. Experimental

### 2.1. Materials and Methods

Fluorine doped tin oxide (FTO) coated glass substrate (Sigma Aldrich, St. Louis, MO, USA) with resistance of 7 Ω/sq was cleaned ultrasonically in acetone, detergent, and ethanol (EtOH) for 15 min and dried. In this study, three types of titanium oxide nanostructures, that is, nanoparticles (NP), nanowire (NW), and nanotubes (NT) in various configurations were tested and TiO_2_ layers were prepared by screen printing method from:

- titania paste consisting of NPs (18 NR-T, Greatcell Solar, Queanbeyan, Australia),
- titania paste consisting of NPs with addition of TiO_2_ NW (3D Nano) (in the ratio of 2.350g NPs paste to 0.005g NWs).
- titania paste consisting of NPs with addition of TiO_2_ NTs (3D Nano) (in the ratio of 3.720 g NPs paste to 0.008g NTs).

A polyester screen (43 T) was used to deposit photoanode with active surface area of 7 mm × 7 mm. The TiO_2_ layers were heated at 500 °C. The prepared TiO_2_ layers on FTO glasses were immersed in N719 (Ru(II)(2,2′-bipyridyl-4,4′-dicarboxylic-acid)(2,2′-bipyridyl-4,4′-ditetrabutylammonium-carboxylate) (NCS)_2_) (Sigma), 3,7′-bis(2-cyano-1-acrylic acid)-10-ethyl-phenothiazine (AC-9 synthesized) or N719 + AC-9 solutions (c = 3 × 10^−4^ M) of dimethylformamide (DMF), chenodeoxycholic acid (CDCA-Sigma) was added to the selected solutions. Synthesis and physicochemical properties of AC-9 compound are described in paper [51]. After 24 h, excess dyes were flushed by EtOH. The fabricated photoanodes were employed to assemble a sandwich-type devices with structure FTO/TiO_2_ + dye/EL-HSE/Pt/FTO. The counter-electrode was nanoplatinum deposited onto FTO glass. The liquid electrolyte (EL-HSE-Sigma), which contains iodide/triiodide redox couple, was placed between the electrodes. 

### 2.2. Measurements

The UV-Vis absorption spectra of dye adsorbed on the TiO_2_ surface were registered using V-570 UV–Vis–NIR Spectrophotometer (Jasco, Inc., Tokio, Japan). The anodes morphology was characterized by atomic force microscopy (AFM) using TopoMetrix Explorer device (Industriële Veiling Eindhoven B.V., CA Eindhoven, The Netherlands), operating in contact mode, in air, in constant force regime. The thicknesses of photoanodes were registered by optical microscope Olympus DSX 510, Olympus, Japan. The photovoltaic parameters such as open-circuit voltage (V_oc_), photocurrent density (J_sc_), fill factor (FF), and power conversion efficiency (PCE) were determined by a PV Solutions Solar Simulator and a Keithley 2400 (under AM 1.5 G, illumination 100 mW/cm^2^) (Tektronix, Inc., Beaverton, OR, USA). The cross-sectional SEM images were taken using a scanning electron microscopy (SEM) Quanta/FEG 250/FEI Co, Thermo Fisher Scientific, Waltham, MA, USA.

The impedance spectra were recorded using BioLogic SP-150 potentiostat, Seyssinet-Pariset, France. All the measurements were performed in the same conditions as the photovoltaic study (AM 1.5 G, illumination 100 mW/cm^2^). The impedance spectra were registered in a potentiostatic mode for a set of applied voltages within the range between 0 V (short-circuit conditions) and 0.7 V (close to open-circuit voltage), with a resolution of 0.05V. The frequency range was from 100k Hz to 0.1 Hz. The amplitude of the alternating current (AC) signal was 10 mV. The analysis of spectra included search for an equivalent electrical circuit and calculation of its parameters according to methodology described in [67,68]. The sets of electrical parameters presented in the paper were obtained from analysis of 176 spectra (15 or 14 spectra for each of 12 samples).

### 2.3. Dye Loading Analysis

The amount of dye adsorbed on TiO_2_ was estimated by adsorption-desorption studies performed according to the literature [17,69,70]. At the beginning, solutions with different concentrations of tested dyes in 10 mM NaOH were prepared and UV-Vis absorption spectra were recorded. Based on registered UV-Vis spectra, a calibration curves for AC-9 and N719 were registered. Further, sensitized TiO_2_ substrates were immersed in 10 mM NaOH solution for 2 h (or even 12 h). During this time, the dye molecules were desorbed from the TiO_2_ films, which was confirmed by the UV-Vis absorption spectra of the substrates. Then, the absorbance of the dye in the NaOH solution (Avantor Performance Materials, Gliwice, Poland) was measured by UV–Vis absorption spectroscopy. The volume of solution was 5 mL and in each case was identical. The dye loading were calculated from a calibration curve based on absorption maxima of reference solutions. However, only N719 dye molecules desorbed from the substrate surface, while the AC-9 dye molecules anchored to TiO_2_ substrates were not completely desorbed even after an extended immersion time.

## 3. Results and Discussion

**Figure 1:**
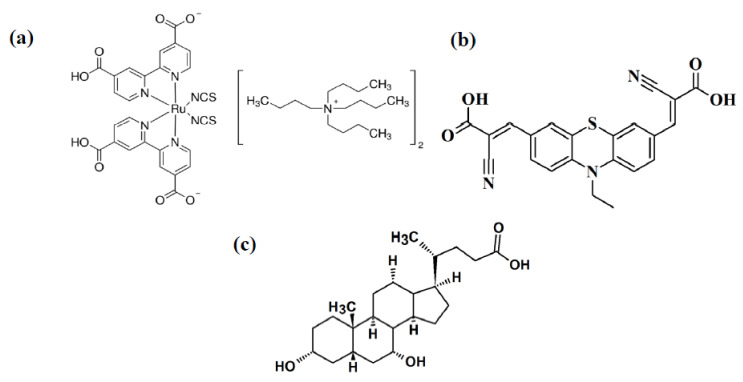
The chemical structures of: (**a**) N719 dyes, (**b**) AC-9, (**c**) CDCA co-adsorbent.

In this paper, the influence of the photoanodes with different TiO_2_ nanostructures and dyes, namely the use of differentiated TiO_2_ nanostructures on photoanode absorption and morphology and finally the basic parameters of dye-sensitized solar cells was studied. Three types of oxide substrates containing only TiO_2_ nanoparticles, TiO_2_ nanoparticles with nanowires, and TiO_2_ nanoparticles with nanotubes were prepared. DSSCs were prepared using two dyes. One of them was commercially available, very often used as a standard dye to reference solar cell fabrication N719. The second one denoted as AC-9 was synthesized as described in our previous work [51]. A device with a mixture of N719 and AC-9 was also prepared, because, co-sensitization, as was mentioned, may improve the photovoltaic performance and allow to reduce the amount of N719. In addition, CDCA as co-adsorbent was added to selected devices. The chemical structures of the used dyes and co-adsorbent are shown in Figure 1.

### 3.1. UV-Vis Absorption of Photoanodes

**Figure 2:**
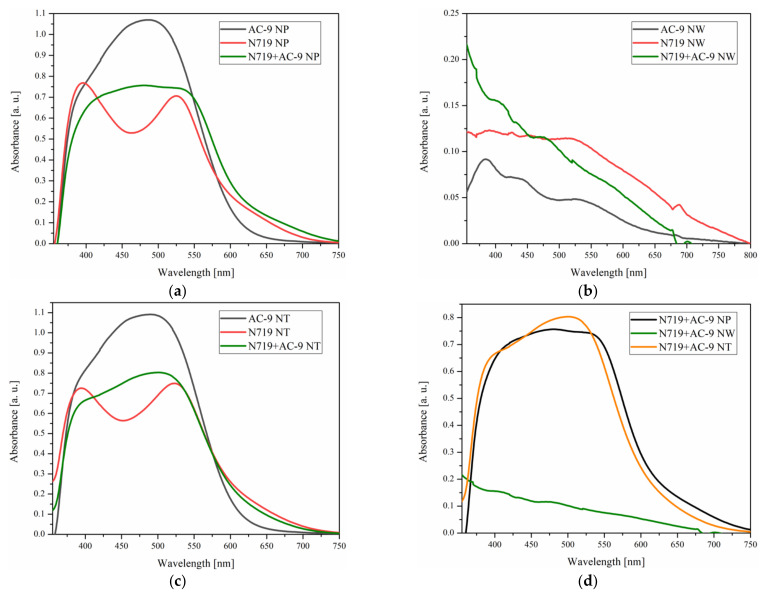
The UV-Vis absorption spectra of tested TiO_2_ substrates consisting of (**a**) only TiO_2_ nanoparticles (NP), (**b**) TiO_2_ nanoparticles with nanowires (NW) and (**c**) nanoparticles with nanotubes (NT); (**d**) different TiO_2_ nanostructural layers containing mixtures of dyes N719 and AC-9.

The optical properties of the prepared photoanodes with different TiO_2_ nanostructures and dyes were evaluated by UV-Vis measurements. The main focus of this work was the absorption range of the photoanodes. The registered spectra of tested photoanodes are presented in Figure 2. In the first three sections, the absorption spectra of different dyes anchored to the same type of substrates (Figure 2a–c) are shown, while Figure 2d presents comparison of the UV-Vis photoanode spectra sensitized with a mixture of N719 and AC-9 on different TiO_2_ layers.

As shown in Figure 2a,c, the absorption spectra of dyes anchored to substrates containing only nanoparticles and nanotubes are very similar. Photoanodes with AC-9 dye containing nanoparticles and nanotubes were characterized by the highest light absorption. However, according to literature [53], this may not always indicate the best photovoltaic performance of solar cells containing a given dye; the wavelength range of dye absorption maximum is also important. It is worth noting that both substrates (NPs and NTs) with N719 and those with dye mixture (N719 + AC-9) are characterized by higher absorption than AC-9 itself in the range from 600 to 730 nm. It seems that in the layer formed from NW, it is difficult for the dye to adsorb to the surface; this was also indicated by only a slight coloration of the oxide layer, which could definitely be reflected in the photovoltaic parameters of the devices. Figure 2d shows the absorption spectra of substrates containing various TiO_2_ nanostructures. As can be seen, the spectra recorded for substrates containing only nanoparticles and nanoparticles with NTs are very similar and well developed, while the spectra for nanoparticles with nanowires differ significantly.

### 3.2. Morphology and Thickness of Photoanodes

The morphology and thickness of the sensitized TiO_2_ layer have a huge impact on the photovoltaic parameters of the prepared dye-sensitized solar cells [50]. The morphology of oxide substrates was examined by atomic force microscopy, determining the RMS parameter, i.e., root-mean-square roughness. RMS parameters were determined for the substrates before immersion in the dye solution as well as with already absorbed molecules of the tested compounds. The thickness of the prepared cells was determined by means of optical microscope and SEM. The results from the optical microscope and AFM are given in Table 1.

As shown in Table 1, the lowest RMS (15–20 nm) values were observed for substrates consisting of TiO_2_ nanoparticles. The addition of NWs or NTs increased the surface roughness of the oxide layer. However, the addition of nanotubes increased the RMS value by about 10 nm. In the case of substrates with NWs, the surface roughness was in the range of 95–150 nm. Therefore, the substrates consisting of only TiO_2_ nanoparticles could be regarded as almost planar. In each case, the photoanode with absorbed dye molecules had a lower RMS value than the TiO_2_ substrate without anchored molecules of the tested compounds. Moreover, it is worth noting that the lowest RMS value was always characterized by the photoanode with an anchored dye AC-9, probably due to smaller particle size of this dye compared to N719, which resulted in better filling of the TiO_2_ pores. Figure S1. shows the AFM micrographs of TiO_2_ substrates without anchored dyes molecules and with adsorbed AC-9 molecules.

Another parameter determined was oxide layer thickness. First, the thickness was determined using an optical microscope. The thickness of the measured TiO_2_ layers with dye molecules was in the range of 8.2–14 µm. It is worth noting that the extreme values, i.e., 8, 2 and 14 µm, occurred only in two layers; the remaining thickness was very similar.

**Figure 3:**
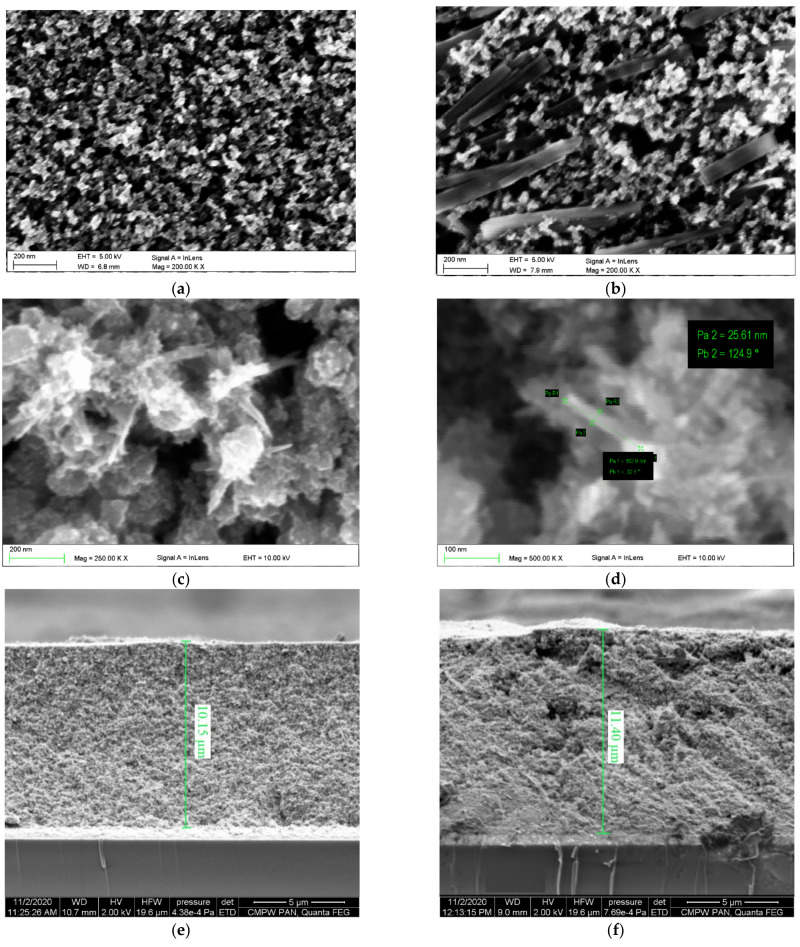

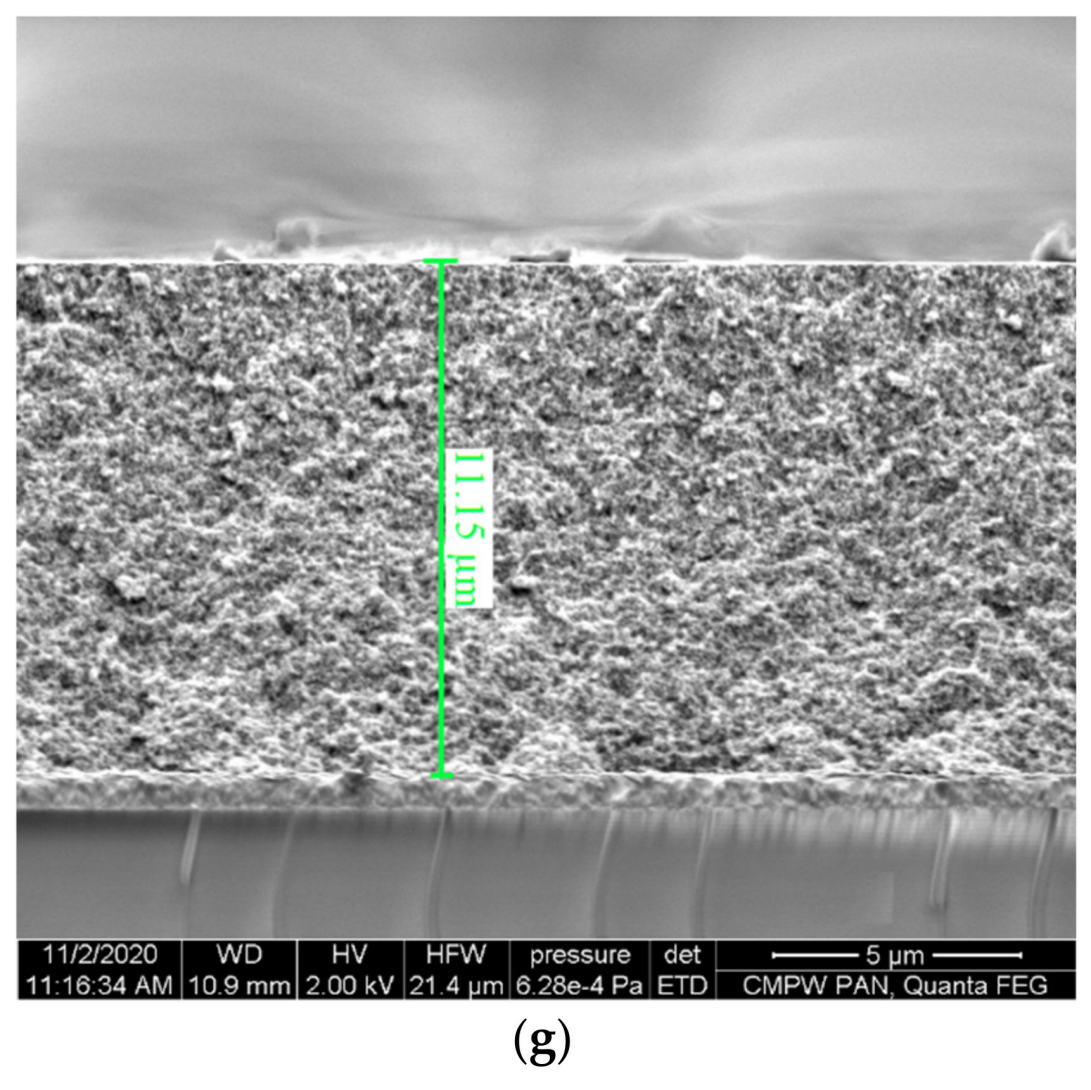
The SEM images of the surface of the TiO_2_ layers containing: (**a**) NPs, (**b**) TiO_2_ NPs-NWs, (**c**,**d**) TiO_2_ NPs-NTs and cross-sectional SEM images of (**e**) TiO_2_ NPs, (**f**) TiO_2_ NPs-NWs and (**g**) TiO_2_ NPs-NTs.

The results of the surface morphology researches of the TiO_2_ layers containing NPs, NPs-NWs and NPs-NTs conducted using scanning electron microscopy (SEM) are shown in Figure 3. The SEM images present a porous structure of the layer composed of TiO_2_ nanoparticles with a regular distribution of pores with a size of 50–100 nm (Figure 3a). The images presenting the surface morphology of NPs-NWs layers (Figure 3b) show nanowires with a diameter of approx. 50 nm and a length of up to 500 nm distributed on the porous surface. However, differences in the length of nanowires are visible. SEM images of NPs-NTs (Figure 3c,d) layers show the porous structure of TiO_2_ nanoparticles with the participation of nanotubes with a length of about 150 nm and a diameter of up to 30 nm. As can be seen, the nanotubes tend to form lumps.

The cross-sectional SEM images of TiO_2_ substrates with nanoparticles, nanowires, and nanotubes without adsorbed dye molecules were taken. The SEM images with designated thicknesses are presented in Figure 3e–g. It was found that the TiO_2_ layers differing in nanostructures exhibited similar thickness 10.15 µm (NP), 11.4 µm (NW), and 11.15 µm (NT).

### 3.3. Photovoltaic Response of DSSCs

The prepared photoanodes were applied for DSSC fabrication with structure FTO/TiO_2_ + dye/EL-HSE/Pt/FTO. The photovoltaic response of devices was evaluated based on current-voltage (J-V) characteristics and electrochemical impedance spectroscopy. Based on analysis J-V curves, the photovoltaic parameters such as open circuit voltage (V_oc_), photocurrent density (J_sc_), fill factor (FF), and power conversion efficiency (PCE) were calculated and are summarized in Table 2. 

Four solar cells differing in the kind of dye were prepared for each type of substrate (NPs, NPs with NWs and NPs with NTs). A similar trend was observed for each type of substrate, i.e., the lowest PCE values were recorded for solar cells containing AC-9 (2.50–4.56%). It is worth mentioning, that these devices were characterized by relatively high photovoltaic parameters as for non-commercial dyes. 

In the presented investigations, one DSSC per kind was prepared, except for device based on N719 and TiO_2_ nanoparticles. In order to confirm the high degree of cell reproducibility, three devices with TiO_2_ nanoparticles sensitized with N719 were fabricated. The small errors of all PV parameters were obtained: ±10 mV, ±0.04 mA/cm^2^, ±0.02 and ±0.01% in the case of V_oc_, J_sc_, FF and PCE, respectively. It can be noticed that the PCE of this device is the same as average efficiency taken over three devices. Taking into account the small errors, which confirm high repeatability of fabricated cells, only one device per kind with TiO_2_ nanotubes and nanowires was prepared, similar as in other articles [54,71].

Each time, the highest PV parameters were obtained for solar cells containing a mixture of two dyes and a co-adsorbent additive (CDCA). The addition of the co-adsorbent to the solution limits the formation of dye aggregates on the surface of TiO_2_ by anchoring the co-adsorbent particles to the oxide surface. The highest PCE value achieved was 6.97% for a solar cell with TiO_2_ substrate with the addition of nanotubes and active layer containing N719, AC-9, and CDCA. When analyzing the influence of substrates, the first thing worth noting is the relatively low photovoltaic parameters for all devices prepared on TiO_2_ substrates with NWs addition. The J_sc_ values were lower comparing to the corresponding solar cells with other substrates (NPs and NTs). It is most likely the formation of a layer on the surface of the photoanode that prevented the anchoring of the dye molecules to the oxide layer. The solar cells prepared on a substrate containing nanotubes showed higher efficiency than their counterparts on other types of substrates. Analyzing individual photovoltaic parameters of these devices, it is worth noting that an increase in current density values was observed. The photocurrent density is related, among other factors, to the amount of adsorbed dye molecules on the oxide substrate. A study was therefore carried out to establish a parameter often referred to as dye-loading. It usually shows the number of dye moles per 1 cm^2^ of TiO_2_ surface. An attempt was made to desorb N719 and AC-9. At the beginning, an immersion time of 2 h in NaOH solution was applied and total desorption of N719 took place (confirmed by UV-Vis measurements), whereas the incomplete desorption of AC-9 was detected due to its limited solubility in NaOH solution. The results of dye loading measurements are given in Table 2. Analyzing dye loading values for individual substrates (NPs, NWs, and NTs), which were 4.04 × 10^−8^, 3.77 × 10^−8^ and 4.45 × 10^−8^ mol/cm^2^, respectively, a correlation with photocurrent density was observed. The smallest amount of absorbed N719 dye molecules was for the NW substrate, which was actually reflected in the J_sc_. The highest dye loading value (4.45 × 10^−8^ mol/cm^2^) was calculated for a solar cell on TiO_2_ substrate with NT and a layer containing N719.

**Figure 4:**
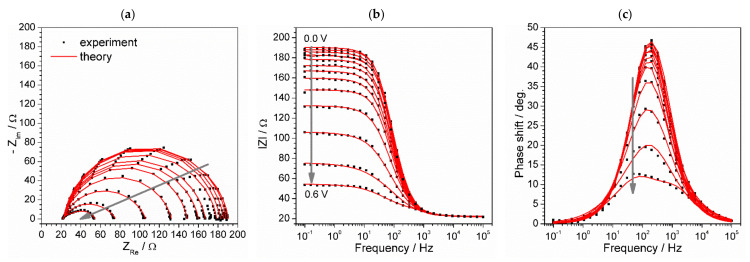
Equivalent circuit fitting results of a set of impedance spectra obtained for a solar cell based on TiO_2_ NPs electrode sensitized with AC-9 dye: (**a**) complex plane (Nyquist) plot, (**b**) impedance module, and (**c**) phase shift dependence on frequency (Bode plot). The grey arrows indicate spectra change with voltage increase.

In next step, electrochemical impedance spectroscopy measurements were carried out. An AC frequency response analysis was applied to determine the resistive and capacitive properties of the electrodes. The typical set of spectra recorded for a DSSC is shown in Figure 4. The impedance, both real (Z_Re_) and imaginary (Z_Im_) components, decreases with voltage. The impedance is maximal under short-circuit conditions (high current and low voltage) and minimal under open-circuit conditions (high voltage and zero current).

**Figure 5:**
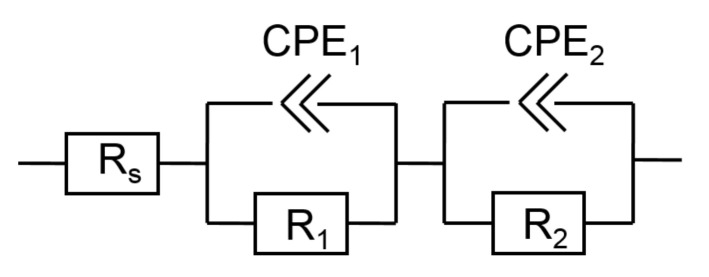
Equivalent electrical circuit of the studied DSSCs. R—resistor, CPE—constant phase element.

The spectra have a form of distorted semicircle. It is well known from the literature that the ideal circle form of the spectrum corresponds to a simple equivalent circuit, including a resistor and a capacitor connected in parallel. However, this is not the case with DSSC. One may spot some deviations from an ideal circle even visually, e.g., by comparing height and half diameter of the arc on the complex plane plot (Figure 4b). Moreover, a slight asymmetry of the phase shift frequency plots suggests the presence of at least two time constants, i.e., at least two couples of resistor and capacitor elements. The search for a model led to an equivalent electrical circuit shown in Figure 5.

The impedance of the CPE element is described by Equation (1).

*Z* = 1/(*Q* × (*j*ω)*^n^*) (1)

where *Q* is quasi-capacitance (F∙sn^–1^), ω—frequency (Hz), *n*—non-ideality factor. When *n* = 1, the expression transforms to the formula for impedance of a capacitor. The formula for the impedance of the model is then described by (2).

(2) $$Z=R_{s}+\frac{1}{\frac{1}{R_{1}}+Q_{1}\times {\left(j\omega \right)}^{n}}+\frac{1}{\frac{1}{R_{2}}+Q_{2}\times {\left(j\omega \right)}^{n}}$$

The circuit has been proven to fit all the collected spectra. The proposed physical meaning of the elements is as follows. The resistor *R_s_* stands for all resisting elements in experimental setup, including electrodes, solution, and connecting cables. Its relatively small value (between 10 and 30 Ω) and absence of dependence on applied voltage are proof of the proposed interpretation.

The two blocks of constant-phase-elements and resistors are equivalents of two electrode-solution interfaces. The common problem in impedance analysis is a correct attribution of each co-block to each particular electrode. The clue to solving this problem is an analysis of obvious differences of electrodes, which a researcher is aware of. Therefore, all the counter-electrodes were the same for all 12 types of studied cells. The repetitive responses may only be observed from the semiconductor electrode.

**Figure 6:**
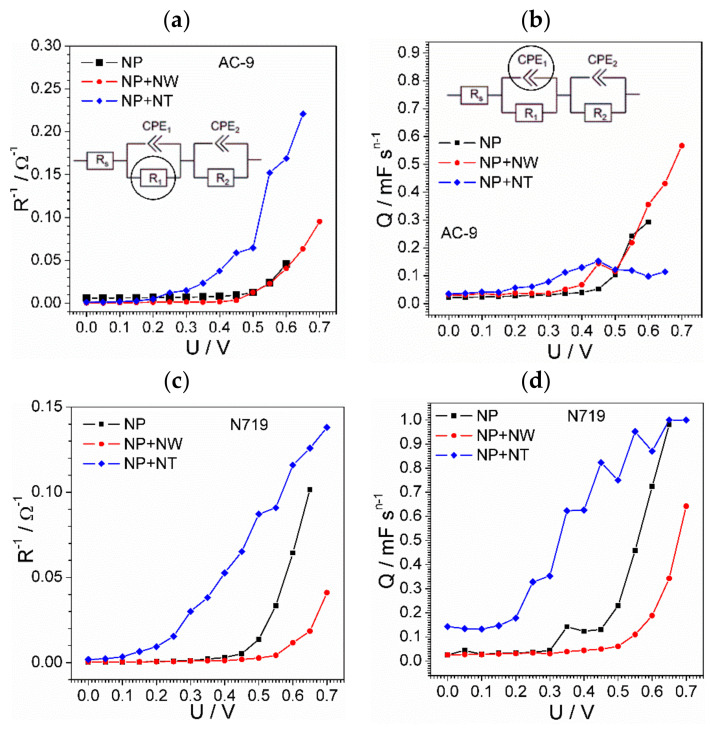

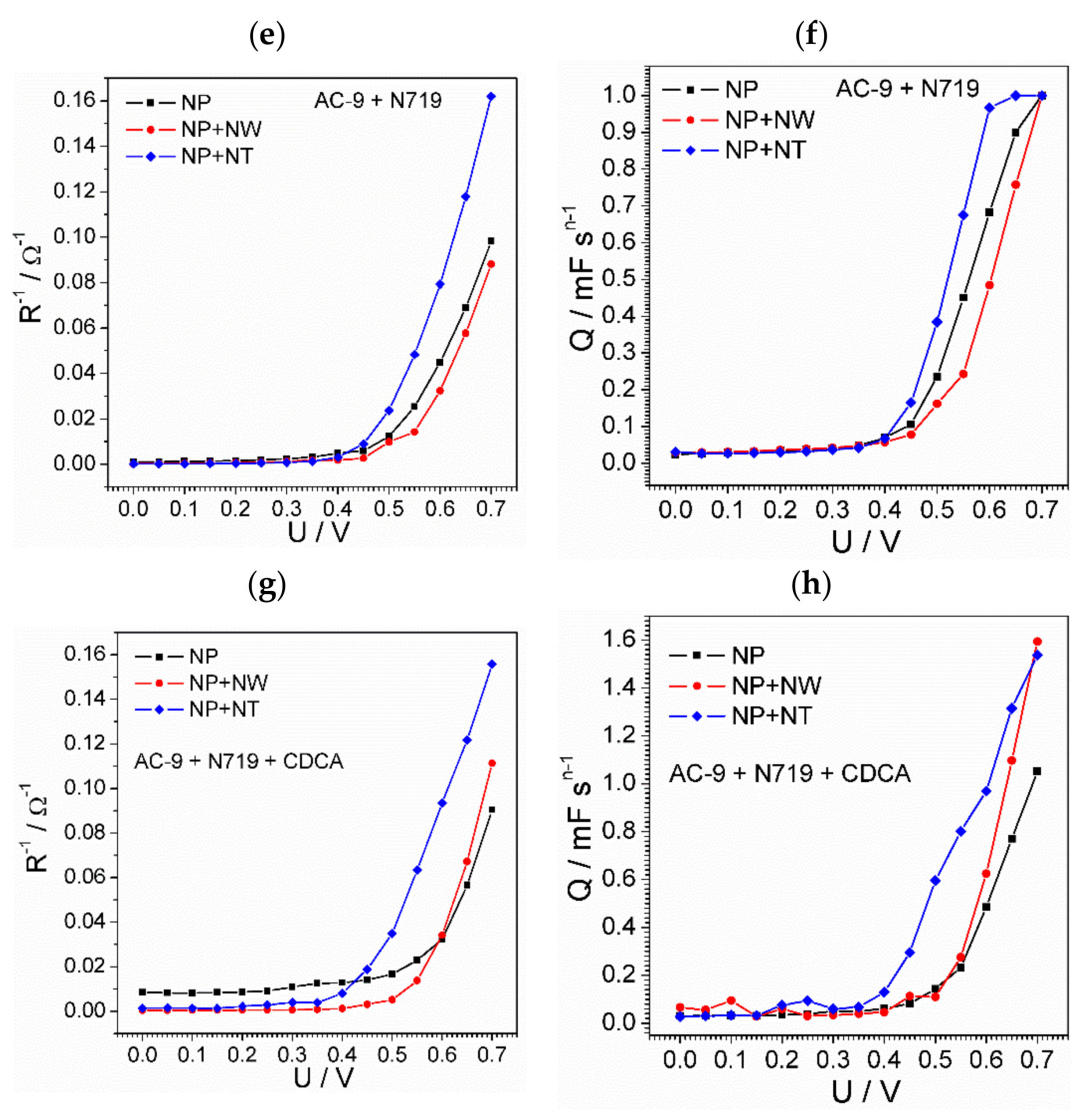
Dependence of TiO_2_-dye electrode resistance (**a**,**c**,**e**,**g**) and capacitance (**b**,**d**,**f**,**h**) on solar cell operating voltage. The results attributed to different TiO_2_ forms are shown by different colors and specified in legend.

All the calculated values of equivalent circuit parameters are given in Figure S2. Taking into account the great number and complexity of the results, here we will discuss the resistive capacitive characteristics of TiO_2_-dye electrodes (Figure 6). The plots are gathered to compare the effect of the semiconductor layer (NP-only nanoparticles, NPs + NTs nanoparticles modified by nanotubes, and NPs + NWs nanoparticles modified with nanowires). The analogous characteristics of the platinum electrode are given in Supporting Information (Figure S2). We show the inverse values of resistance (i.e., conductance), as this value is more representative than the direct value for the following reasons. Firstly, it is the inverse resistance which is expected to correlate with cell performance. Secondly, the values of low resistance can be estimated with higher precision, so that diverging high-resistance results do not complicate the comparison. Many of the demonstrated plots suffer from diverging points, which complicate recognition of the characteristic tendencies. The reason for appearance of such points is long stabilization of the device during study, roughness of the electrode-solution interface, and prehistory of the sample. First of all, it is impossible to assure complete stabilization of the device before registration of each spectrum. The current stabilization takes more than five minutes, which would require more than an hour to measure one device. On the other hand, long measurement time would also cause strong difference in measurement conditions between the first and last spectra. Another reason arises from mathematical analysis of the spectra. The seven parameters of the model (one for each of three resistors and two for each of two CPEs) make the model extremely sensitive to the weak noise of the results. Nevertheless, the particular tendencies have been revealed. We decided not to embellish the data by removing obviously anomalous points to present to the reader a realistic picture of the impedance results.

To describe charge transfer change with cell voltage, one has to consider basic equations of electrochemical kinetics. The current passing through the photoanode-solution interface is proportional to the concentration of reduced species at the electrode surface *c*_Red,S_ (i.e., I^−^ species) and is determined by oxidation rate constant (3).

(3) $$i=k_{Ox}c_{Red,S}$$

Despite a very low distance between the electrodes, the surface concentration may differ from the bulk concentration, especially when the cell operates in a high current mode. The charge-transfer resistance (*R_ct_*), whose value is obtained from impedance spectrum analysis, indicates how strong voltage modulation affects the current (4).

(4) $$R_{ct}^{-1}=\frac{\partial i}{\partial E}=c_{Red,S}\frac{\partial k_{Ox}}{\partial E}+k_{Ox}\frac{\partial c_{Red,S}}{\partial E}$$

The first derivative is an exponential function, which is obtained from the Butler-Volmer equation. The second term is much more complicated to be derived theoretically. If the charge transfer process is slow (much slower than diffusion, to be more exact), then the surface concentration remains equal to the bulk concentration within the wide potential range, so the second term of (4) equals zero. This is the case when a cell operates at a small current. In the case of a fast redox process, the surface concentration is determined by diffusion and could be obtained theoretically by solving Fick’s diffusion law.

**Figure 7:**
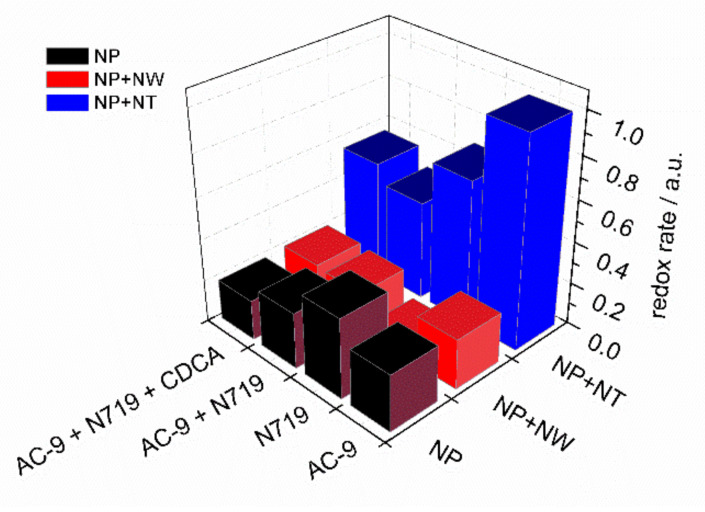
Comparison of the charge transfer rates for 12 types of studied cells. The data is based on inverse charge transfer resistance values measured at 0.6 V.

The complexity of theory and non-ideality of the real objects do not allow to conduct the complete kinetical analysis of the processes. However, the obtained *R^ct−^*^1*−U*^ dependences (Figure 6a,c,e,g) suggest that the photoanode process is limited by charge-transfer kinetics. Concerning the activity of substrates, Figure 7 reveals the leading position of the nanotube-modified electrode (NT). The rates of charge transfer for three substrates and four combinations of dyes are compared in Figure 7.

Another parameter, pseudo-capacitance, is defined not only by redox kinetics, but also by reversible electrosorption of the charged species. Thus, this parameter can be used for a rough estimation of the active surface area. The correlation between inverse charge-transfer resistance and capacitance implies that the efficiency of the cells employing nanotube-modified electrodes over other types of cells is conditioned by facile charge transfer as well as developed surface area. 

## 4. Conclusions

The series of dye-sensitized solar cells consisting of different TiO_2_ substrates, such as nanoparticles, nanoparticles with nanotubes, and nanoparticles with nanowires and dyes (N719, AC-9) were prepared. The studies carried out on the optical properties of the prepared photoanodes showed that the highest absorption was found for the substrate containing the addition of TiO_2_ nanotubes for all the dyes tested. It is worth noting that the presence of nanowires made it difficult to adsorb dye molecules to the substrate, as shown by the UV-Vis tests. The number of dye molecules (N719) anchored to the oxide substrate was also found, the highest value of the dye-loading was recorded for the substrate with the addition of nanotubes (4.45 × 10^−8^ mol·cm^−2^) and the smallest for the substrate with the addition of nanowires (3.77 × 10^−8^ mol·cm^−2^). The RMS values of tested substrates with addition of NP or NT were 20 and 35 nm, respectively, while the substrate containing NW addition showed a much higher RMS value (150 nm). In each case, the presence of dye molecules on the oxide surface reduced the RMS value, smoothening it out. Considering the photovoltaic performance of fabricated DSSCs, it can be concluded that the solar cells prepared on a substrate with TiO_2_ nanotubes addition showed the best PV response. Moreover, it is worth noting that the cell sensitized with a mixture of dyes (N719 and AC-9) and CDCA additive exhibited the highest efficiency (6.97%). The individual capacitive-resistive characteristics of each electrode were extracted from impedance spectra of the cells. Analysis of the voltage dependences of the obtained parameters allowed for relative comparison of the rates of the current-generating redox processes at different materials. The nanotube-nanoparticle modified titanium oxide was shown to be the most effective electrode probably due to high active surface area, which can be confirmed by dye loading values being the highest and facile charge transfer. However, the impact of NT on charge transfer is an assumption, which, in this case, has not been yet experimentally confirmed.

## Figures and Tables

**Table 1:** Thickness and roughness parameters of TiO_2_ layers.

TiO_2_ Nanoparticles	Sensitizers	AFM	Optical Microscope	SEM
		RMS (nm)	Thickness (µm)	Thickness (µm)
Nanoparticles	-	20	-	10.15
	N719	16	10.3	-
	AC-9	15	14	-
	N719 + AC-9	16	9.5	-
	N719 + AC-9 + CDCA	18	10.7	-
Nanoparticles/Nanowires	-	150	-	11.4
	N719	135	12.5	-
	AC-9	95	10.3	-
	N719 + AC-9	130	10	-
	N719 + AC9 + CDCA	138	11.1	-
Nanoparticles/Nanotubes	-	35	-	11.15
	N719	30	11.3	-
	AC-9	28	9.5	-
	N719 + AC-9	30	11.4	-
	N719 + AC-9 + CDCA	33	8.2	-

**Table 2:** Photovoltaic parameters of fabricated solar cells.

TiO_2_ Nanostructure	Compounds	V_oc_ (mV)	J_sc_	FF	PCE (%)	Dye Loading
			(mA cm^−2^)	(-)		(mol cm^−2^)
Nanoparticles	N719	720	15.8	0.44	5.1	4.04 × 10^−8^
	AC-9	675	10.54	0.58	4.21	-
	N719 + AC-9	730	15.06	0.54	6.1	-
	N719 + AC-9 + CDCA	732	15.2	0.59	6.69	-
Nanoparticles/Nanowires	N719	738	8.89	0.62	4.15	3.77 × 10^−8^
	AC-9	679	5.9	0.61	2.5	-
	N719 + AC-9	740	11.48	0.57	4.9	-
	N719 + AC-9 + CDCA	740	11.34	0.63	5.44	-
Nanoparticles/Nanotubes	N719	725	16.27	0.46	5.56	4.45 × 10^−8^
	AC-9	678	10.71	0.61	4.56	-
	N719 + AC-9	714	16.33	0.54	6.48	-
	N719 + AC-9 + CDCA	711	16.6	0.58	6.97	-

## Data Availability

The data presented in this study is available upon request of the respective author. The data can serve as a basis for initiating a patenting process. Later, they can be deposited and made available in open data repositories (e.g., Zenodo or RepOD).

## Supplementary Materials

The following are available online at https://www.mdpi.com/article/10.3390/ma14071633/s1.
