# Supplementary material for: Impact of TiO2 Nanostructures on Dye-Sensitized Solar Cells Performance
Source: Materials (Basel). 2021 Mar 26;14(7):1633. doi: 10.3390/ma14071633 (PMC8036646; doi:10.3390/ma14071633)
Supplement: Supplementary file 1 [file materials-14-01633-s001.pdf]

materials

Supplementary Materials

Impact of TiO_2 Nanostructures on Dye-Sensitized Solar Cells Performance

Paweł Gnida [1], Paweł Jarka [2], Pavel Chulkin [3], Aleksandra Drygała [2], Marcin Libera [4], Tomasz Tański [2,*] and Ewa Schab-Balcerzak [1,2,*]

[1] Centre of Polymer and Carbon Materials, Polish Academy of Sciences, 34 M. Curie-Skłodowska Str., 41-819 Zabrze, Poland; pgnida@cmpw-pan.edu.pl
[2] Institute of Engineering Materials and Biomaterials, Silesian University of Technology, 18a Konarskiego Str., 44-100 Gliwice, Poland; pawel.jarka@polsl.pl (P.J.); aleksandra.drygala@polsl.pl (A.D.)
[3] Faculty of Chemistry, Silesian University of Technology, 9 Strzody Str., 44-100 Gliwice, Poland; pavel.chulkin@polsl.pl
[4] Institute of Chemistry, University of Silesia, 9 Szkolna Str., 40-007 Katowice, Poland; marcin.libera@us.edu.pl
* Correspondence: tomasz.tanski@polsl.pl (T.T.); ewa.schab-balcerzak@us.edu.pl (E.S.-B.)

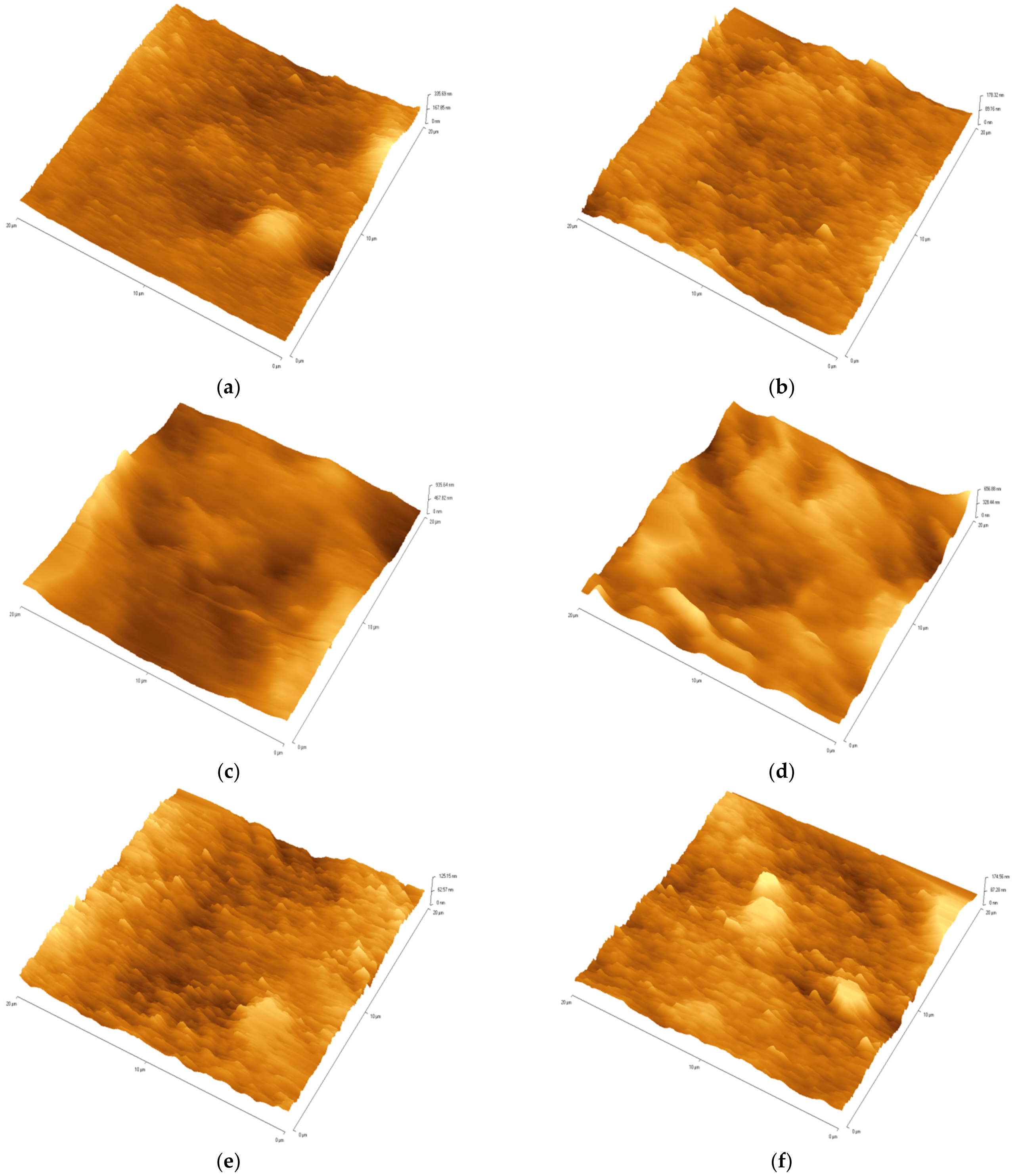

Figure S1. The AFM micrographs of (**a**) NP TiO2 without dye, (**b**) NP TiO2 with AC-9, (**c**) NW TiO2 dye, (**d**) NW TiO2 with AC-9, (**e**) NT TiO2 dye, (**f**) NT TiO2 with AC-9.

Impedance spectra analysis results

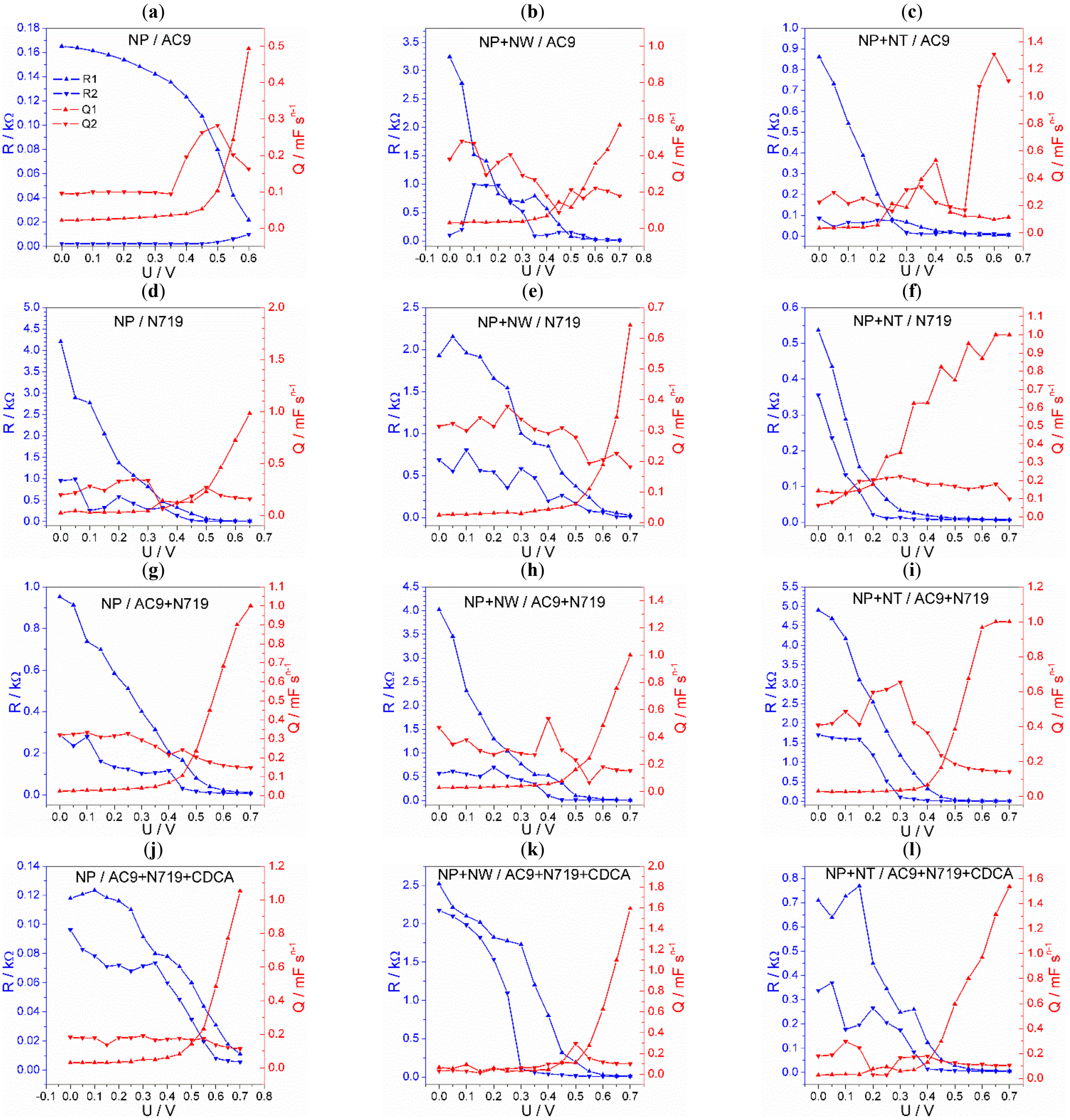

Figure S2. Values of electrode resistances (blue points) and capacitances (red points) calculated from impedance spectra. Table . he columns correspond to different substrates, the rows correspond to different dyes. A common legend is given in (**a**).

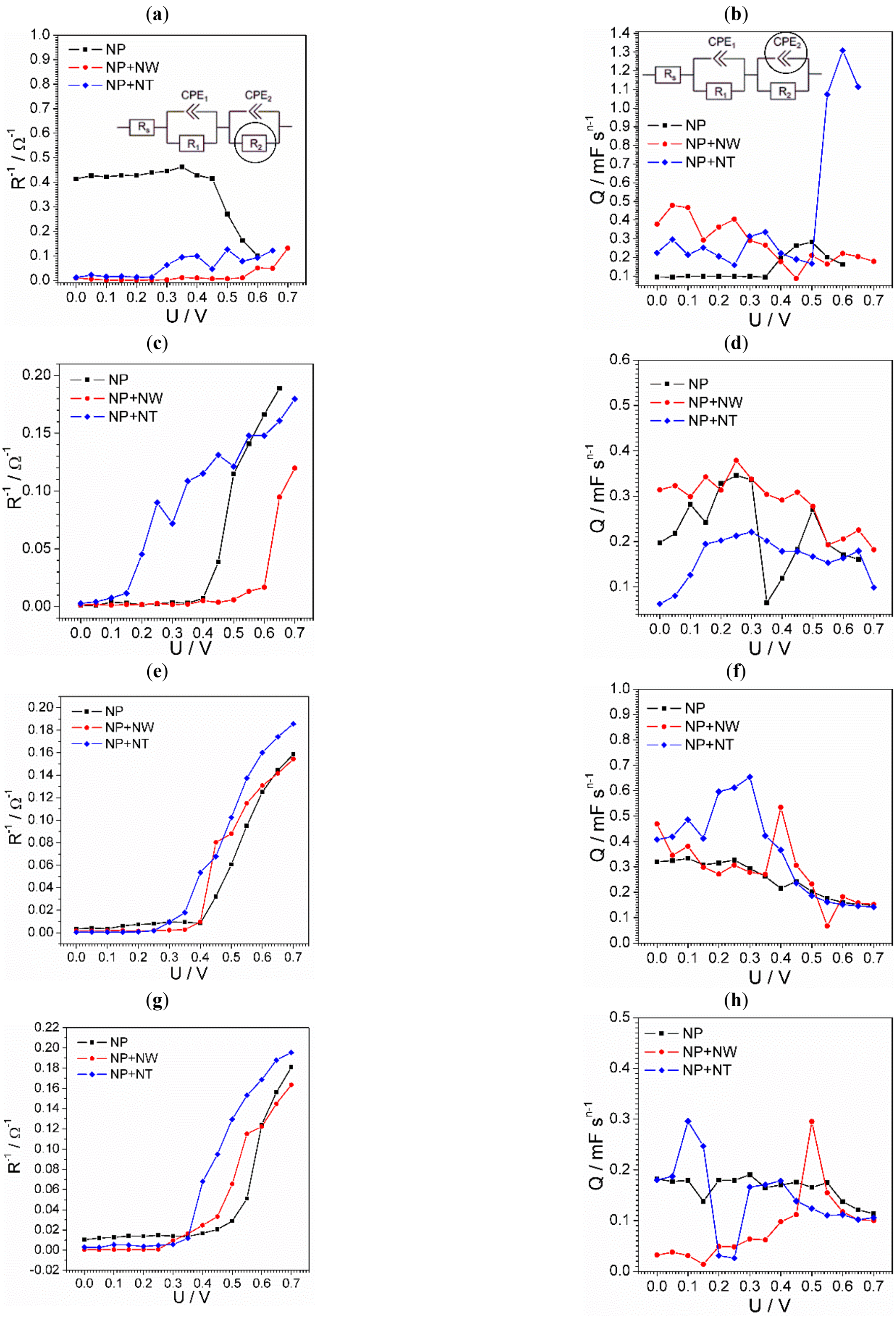

Figure S3. Dependence of platinum electrode inverse resistance (left column) and capacitance (right column) on cell operating voltage for different dyes: AC-9 (**a**,**b**), N719 (**c**,**d**), mixture of AC-9 and N719 (**e**,**f**), mixture of AC-9, N719 and CDCA (**g**,**h**).
